# Intersubject correlations in reward and mentalizing brain circuits separately predict persuasiveness of two types of ISIS video propaganda

**DOI:** 10.1038/s41598-024-62341-3

**Published:** 2024-06-12

**Authors:** Michael S. Cohen, Yuan Chang Leong, Keven Ruby, Robert A. Pape, Jean Decety

**Affiliations:** 1 Department of Psychology, University of Chicago, 5848 S. University Ave, Chicago, IL 60637 USA; 2 Department of Political Science, University of Chicago, Chicago, IL USA; 3 Department of Psychiatry and Behavioral Neuroscience, University of Chicago, Chicago, IL USA; 4 Chicago Project on Security and Threats, University of Chicago, Chicago, IL, USA

**Keywords:** Human behaviour, Social neuroscience

## Abstract

The Islamist group ISIS has been particularly successful at recruiting Westerners as terrorists. A hypothesized explanation is their simultaneous use of two types of propaganda: Heroic narratives, emphasizing individual glory, alongside Social narratives, which emphasize oppression against Islamic communities. In the current study, functional MRI was used to measure brain responses to short ISIS propaganda videos distributed online. Participants were shown 4 Heroic and 4 Social videos categorized as such by another independent group of subjects. Persuasiveness was measured using post-scan predictions of recruitment effectiveness. Inter-subject correlation (ISC) was used to measure commonality of brain activity time courses across individuals. ISCs in ventral striatum predicted rated persuasiveness for Heroic videos, while ISCs in mentalizing and default networks, especially in dmPFC, predicted rated persuasiveness for Social videos. This work builds on past findings that engagement of the reward circuit and of mentalizing brain regions predicts preferences and persuasion. The observed dissociation as a function of stimulus type is novel, as is the finding that intersubject synchrony in ventral striatum predicts rated persuasiveness. These exploratory results identify possible neural mechanisms by which political extremists successfully recruit prospective members and specifically support the hypothesized distinction between Heroic and Social narratives for ISIS propaganda.

## Introduction

During the past decades, the Islamic State of Iraq and Syria (ISIS) has been uniquely successful at recruiting Western-born Muslims and converts to Islam to join their cause^1,2^. A central pillar of ISIS’s recruitment strategy has been its online propaganda, especially videos produced by the group in English and other Western European languages and featuring fighters from North America and Europe. Indeed, the vast majority of individuals indicted in the U.S. for supporting ISIS are American-born (61%), watched ISIS videos, and self-reported videos in their radicalization (85%)^1^. Understanding what makes ISIS video propaganda effective in enhancing support for the group among diverse Western audiences advances the science of persuasion and political mobilization and also contributes to countering the appeal of extremist groups.

One potential reason for ISIS’s success in the West is the use of a “Heroic martyr” narrative alongside the “Social martyr” narrative more typical for terrorist propaganda. As research documents, ISIS Heroic narratives are analytically distinct from and occur more frequently in ISIS Western-directed video propaganda than Social narratives^3^. Specifically, the Social martyr narrative features “good Muslims” who gain recognition for fulfilling their religious obligation to protect embattled communities based on strong social ties to those communities. The Heroic martyr narrative, in contrast, casts its heroes as ordinary individuals who discover their true potential through extraordinary action, trading on tropes common to Western literature and action movies more than religious exegeses and sincerity of belief. The use of a traditional (the Social) and “westernized” (the Heroic) narrative has the potential to extend ISIS’s reach among Muslims in the West who relate differently to Islam and life in the West.

Heroic narratives have been found to be particularly effective at appealing to people with weak ties to embattled Muslim communities while Social narratives appeal to those with stronger ties to such communities. A survey study found that American-born converts to Islam and Americans born into Muslim families in the U.S. rated Heroic videos as more persuasive than did immigrants from Muslim countries^4^. Additionally, when reviewing court records of actual ISIS members who were charged criminally in the United States for their terrorist activities, converts to Islam were scored as more likely to cite motivations in line with Heroic narratives while those born into Muslim families were more likely to indicate motivations in line with Social narratives^4^. Finally, an analysis of ISIS propaganda found that Western-directed propaganda from ISIS includes a mix of Social and Heroic narrative framings^4^. A key hypothesis following from these data is that ISIS may enhance its recruitment strategy success by choosing to rely on a mix of these narrative types specifically because they appeal to potential recruits in different ways.

One study using high-density electrophysiology (EEG) provided initial evidence that ISIS propaganda videos evoke distinct neural mechanisms based on whether they rely on a Heroic or Social narrative framing^5^. Most notably, Heroic videos were associated with increased frontal beta power, interpreted as reflecting greater personal relevance and positive expectations. In contrast, Social videos were associated with reduced alpha power, reflecting greater engagement of attention for these videos, and greater frontal theta power, which could imply stronger emotion regulation. Together, these initial findings provided support for the theorized distinction between Heroic and Social videos^3^. The present work builds on these findings by using fMRI rather than EEG to measure brain activity in participants exposed to these propaganda videos. Functional MRI has advantages relative to EEG in its ability to access the entire brain, including deep structures, and in providing more precise spatial localization of effects.

The same study also examined behavioral ratings of stimulus features and differences between individuals that modulate the effectiveness of ISIS propaganda videos. One feature typically shown to modulate persuasiveness is narrative transportation, which is the degree to which people can imagine themselves in the narrative and pay close attention to it, e.g.,^6^. Prior work confirmed that narrative transportation predicted persuasiveness in an overlapping set of videos as that used in the present study, with a stronger relationship in men than in women^5^. Another factor that this prior work suggested may be relevant is participants' religiosity. Specifically, in a multivariate decoding analysis of EEG data, male participants who reported a higher degree of religious observance showed a greater difference in brain response for Heroic vs. Social videos than did male participants who were less religiously observant. Narrative transportation and religiosity measures were acquired for participants in the present study as well. Finally, justice sensitivity, a personality trait that captures how people react to injustices^7–10^, was examined for its possible relevance to rated persuasiveness of ISIS recruitment videos in the present study. This personality measure, some components of which are related to empathy^11^, has been associated with extremist political beliefs in Western contexts. For instance, higher sensitivity to injustices where one is personally victimized, and lower sensitivity to injustices where others are victimized, were associated with greater support for both Donald Trump in the United States and for the far-right AfD party in Germany^12^.

### Neuroeconomics and neuroforecasting approaches

Past studies from the neuroeconomics and neuroforecasting literatures help to motivate the data analysis strategy used in the present work. Specifically, brain responses are used to predict behavioral ratings over and above behavioral predictors. There are many relevant studies showing how increased brain activation in response to discrete stimuli can predict choices better than behavioral measures. Activity in the reward/valuation system, particularly in the ventral striatum (VS)/nucleus accumbens (NAcc) and to a somewhat lesser extent in medial prefrontal cortex (mPFC), is greater in response to things that people choose, prefer, or like. This result is found in a variety of domains including facial attractiveness^13^, consumer products^14^, political candidates^15^, newspaper articles^16^, crowdfunding campaigns^17^, and YouTube videos^18^. These reward responses can be modulated by the opinion of others^19^ and social feedback^20^. Additionally, reward responses among a small sample of participants in the scanner can predict popularity of the same stimuli in a larger population (e.g.,^16–18^).

There is evidence that activity in the mentalizing circuit of the brain can also predict persuasiveness and attitude change. Note that ventral mPFC is active in mentalizing tasks (see, e.g.,^21^) in addition to playing a key role in processing reward^22^, but dorsal mPFC is more exclusively associated with social information processing. As has been noted in a prior review ^23^, studies of persuasion have diverged in emphasizing findings from dorsal vs. ventral regions of mPFC. Still, dorsomedial PFC (dmPFC) and other key brain regions that process social information have been shown to be more active when processing more persuasive stimuli. In one study^24^, text and video messages that participants rated as more persuasive, about a range of topic areas and across cultures, produced increased activity in dmPFC and bilaterally in posterior superior temporal sulcus (pSTS) and temporal pole (TP) relative to stimuli labeled as less persuasive. In addition, while many of the neuroforecasting studies that examine messaging effectiveness in the context of smoking cessation have focused on ventral mPFC ROIs, others have found critical effects in dorsal mPFC. This includes one study using messaging specifically tailored to participants’ values and interests^25^, and another that identified brain regions via an interaction between content and format that follows from the elaboration likelihood model of persuasion^26^. In both of the preceding studies, activity in dmPFC correlated with effectiveness of smoking cessation programs within the sample. Additionally, click-through rate of online anti-smoking ads in a large population was predicted by brain activity in a small scanned sample in brain regions including dmPFC, vmPFC, and L TPJ, as well as in posterior cingulate (PCC), a critical region for introspective processing, specifically in response to negatively-valenced smoking-relevant messaging^27^. Finally, in a different task domain, adolescents have been found to activate mentalizing regions when processing ratings from both parents and peers regarding the aesthetic value of art, and the degree to which these regions increase in activity corresponds to how much ratings tend to shift from a prescan baseline to match others’ ratings^28^.

Some work has begun to examine the relationship between reward and mentalizing in persuasion. One study found that both reward and mentalizing circuits were activated when people changed their ratings of hypothetical video games in response to feedback from other people, but the degree of rating change was *negatively* correlated with functional connectivity between these networks^29^. This result suggests that the two networks make separable contributions to persuasion. More specifically, reward activity appeared to represent the value of the information regardless of its valence, while mentalizing activity tended to be stronger when ratings were impacted by negatively-valenced information. A different relationship between reward and mentalizing networks emerges from the value-based virality model, in the related domain of online content sharing. This work suggested an indirect effect by which increased activity in mentalizing and self-related processing regions of the brain leads to increased reward system activity, which then leads to greater content sharing^16^. Thus, reward/valuation and mentalizing systems can work either independently or in concert depending on situational factors.

### Inter-subject correlations

One important methodological difference between the current study and most prior work is that we examine inter-subject correlations (ISCs) in the brain. This method more fully leverages the data available with naturalistic video stimuli, relative to more traditional analyses^30^. Specifically, ISC focuses on dynamic changes in brain activity over time, rather than measuring the amplitude of brain activity in response to a specific stimulus. Applying an ISC analysis to the time course of activity for an individual in a given brain region, versus the average time course for all other participants in that same region, identifies the degree to which processing in that region is stimulus-driven^31^. The response must also be shared across individuals, and not idiosyncratic, to drive an increase in ISCs estimated using this approach^32^.

Some prior studies have related ISCs to engagement with and popularity of video stimuli. For instance, EEG measures of neural synchrony while viewing a television show have been positively correlated with real-world minute-to-minute Nielsen ratings and with which scenes in the show produced greater engagement on Twitter^33^. Similarly, neural synchrony measured via EEG while viewing a movie trailer can predict real-world ticket sales, and this measure predicts real-world popularity better than traditional behavioral measures from the same sample^34^. ISCs computed based on fMRI BOLD signal have also been associated with such real-world outcome measures. For instance, ISC in brain regions associated with mentalizing—temporal pole and TPJ, an emotion-related region of cerebellum, and, in one experiment, dmPFC—have been associated with out-of-sample liking ratings for commercials and movie trailers^35^. Higher rated persuasiveness of anti-alcohol public service announcements (PSAs) has similarly been associated with ISCs in mentalizing regions such as dmPFC and precuneus, as well as in visual cortex, insula, inferior prefrontal gyrus, and supramarginal gyrus^36^. A study in which participants listened to political speeches, previously rated on how powerful they were perceived to be, found that more powerful speeches led to stronger ISCs in mPFC and in superior temporal gyrus^37^. Finally, meaningful personal narratives from the series “This I Believe” evoked greater ISCs in the mentalizing circuit, particularly dmPFC, TPJ, and precuneus, compared to a non-personal VCR instruction manual text control^38^. Together, this literature supports the idea that stimuli that evoke more consistent fluctuations in brain activity are more popular, persuasive, and yield greater engagement than those that evoke more variable activity timecourses. Such results are often observed specifically in brain regions associated with mentalizing.

In the exploratory analyses that follow, ISCs in brain regions associated with reward and mentalizing are associated with persuasiveness ratings. There is a double dissociation, however, as persuasiveness of Heroic videos is modulated primarily by ISCs in a ventral striatum region of interest (ROI)^22^, while persuasiveness of Social videos is modulated by mean ISC across a network of brain regions associated with mentalizing, particularly dmPFC^21^. Aggregate differences in ISCs between Heroic and Social stimuli are also apparent. These results together support the hypothesized distinction between videos relying on a Heroic narrative and those relying on a Social narrative.

## Results

Each participant was presented with four Heroic videos and four Social videos in the MRI scanner, with one video of each type presented per scan run in randomized order. Full neuroimaging data were acquired from 46 participants, but the post-scan questionnaire was only presented to the final 34 participants. In this questionnaire, participants re-watched and rated each video on measures of persuasiveness and narrative transportation. Data from the larger sample (n = 46) were averaged to compute group-level time courses to which each individual’s data were compared, with the comparison individual always excluded from this average. Final results, however, are only reported from the subset (n = 34) who completed all behavioral measures.

### Regression analyses—behavioral measures

Persuasiveness was used as the dependent measure for all regression analyses. It was operationalized as participants’ ratings on a 1–7 scale to the following question: “This video would help the militant group recruit,” collected after the functional MRI session about each video. All mixed-effects models were computed with the *lme4* and *lmerTest* packages in R using maximum likelihood estimation. Gender was coded such that gender = 0 for males, gender = 1 for females, and gender = 0.5 for one non-binary participant. Stimulus types were coded such that Social = 0 and Heroic = 1. These dichotomous/ordinal variables were then standardized, along with all continuous variables, to compute standardized β coefficients. An initial linear mixed-effects model including clip type (Heroic or Social) and participant gender, with a random effect for intercept between participants, showed a main effect of clip type (β = 0.252, *t* = 4.52, *p* < 0.001) on persuasiveness, with Heroic stimuli rated as more persuasive, and no main effect of participant gender (β = − 0.036, *t* = − 0.46, *p* = 0.65) or interaction with gender (β = 0.055, *t* = 1.00, *p* = 0.32).

Participants were also asked to rate each clip post-scan on three questions that together constituted a measure of narrative transportation. Prior to the scan session, each individual had responded to measures of Justice Sensitivity (4 subscales)^39^ and Religiosity (3 subscales)^40^. In prior work^5^, some relationships between behavioral variables and persuasiveness interacted with participant gender and/or with stimulus type. To determine which behavioral/demographic measures predicted rated persuasiveness, a series of linear mixed-effects models were run in which each variable and its interaction with participant gender and stimulus type were separately regressed as fixed effects on persuasiveness, with intercept for each participant modeled as a random effect. Across these models, only two behavioral variables showed a significant relationship with persuasiveness: Narrative Transportation and the Perpetrator subscale of Justice Sensitivity. Separate models for each of these variables are reported in Supplemental Material. A combined model that included both sets of variables yielded a lower AIC (701.47) than the model with only Narrative Transportation (713.17) or only Justice Sensitivity Perpetrator subscale (737.35); thus, this was chosen as the best behavioral model (Table 1).

**Table 1:** Behavioral model predicting rated persuasiveness (n = 269 observations in 34 participants).

	β	t	p
(Intercept)	− 0.026	− 0.359	0.72
Participant Gender (Male = 0, Female = 1)	− 0.066	− 0.91	0.37
Stimulus Type (Social = 0, Heroic = 1)	**0.285**	**5.52**	< 0.001 ***
Justice Sensitivity (Perpetrator subscale)	0.102	1.24	0.22
Narrative Transportation	**0.350**	**6.16**	< 0.001 ***
Participant Gender x Stimulus Type	**0.104**	**2.04**	0.043*
Justice Sensitivity (Perpetrator subscale) x Participant Gender	0.150	1.95	0.060 ~
Justice Sensitivity (Perpetrator subscale) x Stimulus Type	− **0.229**	− **3.95**	< 0.001 ***
Narrative Transportation x Participant Gender	0.017	0.31	0.76
Narrative Transportation x Stimulus Type	**0.151**	**2.90**	0.004**
Justice Sensitivity (Perpetrator subscale) x Participant Gender x Stimulus Type	− 0.017	− 0.31	0.75
Narrative Transportation x Participant Gender x Stimulus Type	**0.118**	**2.32**	0.021*

Significant values are in bold.

To further examine the interactions in behavioral effects shown in Table 1, these effects were broken down by gender and clip type. For male participants, there was a positive main effect of narrative transportation on persuasiveness (β = 0.360, *t* = 4.12, *p* < 0.001) and no interaction between narrative transportation and clip type (β = 0.011, *t* = 0.14, *p* = 0.89). Thus, greater narrative transportation predicted higher persuasiveness ratings in males regardless of stimulus type. For the Justice Sensitivity Perpetrator subscale, men showed an interaction with clip type (β = − 0.256, *t* = − 2.75, *p* = 0.007) with no main effect (β = − 0.112, *t* = -0.85, *p* = 0.41). Breaking the analysis down by clip type showed that men with a higher Justice Sensitivity Perpetrator score rated Heroic clips as less persuasive (β = − 0.387, *t* = − 2.41, *p* = 0.030), but no such relationship was apparent for Social clips (β = 0.148, *t* = 1.00, *p* = 0.32). In contrast, female participants showed both a main effect of narrative transportation (β = 0.353, *t* = 4.79, *p* < 0.001) and an interaction between narrative transportation and clip type (β = 0.240, *t* = 3.55, *p* < 0.001). Breaking the analysis down by clip type showed that in women, higher narrative transportation predicted greater persuasiveness for Heroic clips (β = 0.643, *t* = 6.93, *p* < 0.001) but not Social clips (β = 0.112, *t* = 1.00, *p* = 0.32). For the Justice Sensitivity Perpetrator subscale, women showed an interaction with clip type (β = − 0.179, *t* = − 2.95, *p* = 0.004) and a marginal main effect (β = 0.165, *t* = 2.05, *p* = 0.055). Higher Justice Sensitivity Perpetrator scores were associated in women with higher persuasiveness ratings for Social clips (β = 0.325, *t* = 2.80, *p* = 0.011) but not for Heroic clips (β = − 0.016, *t* = − 0.17, *p* = 0.86).

### Predicting persuasion by reward-related brain activity

**Figure 1:**
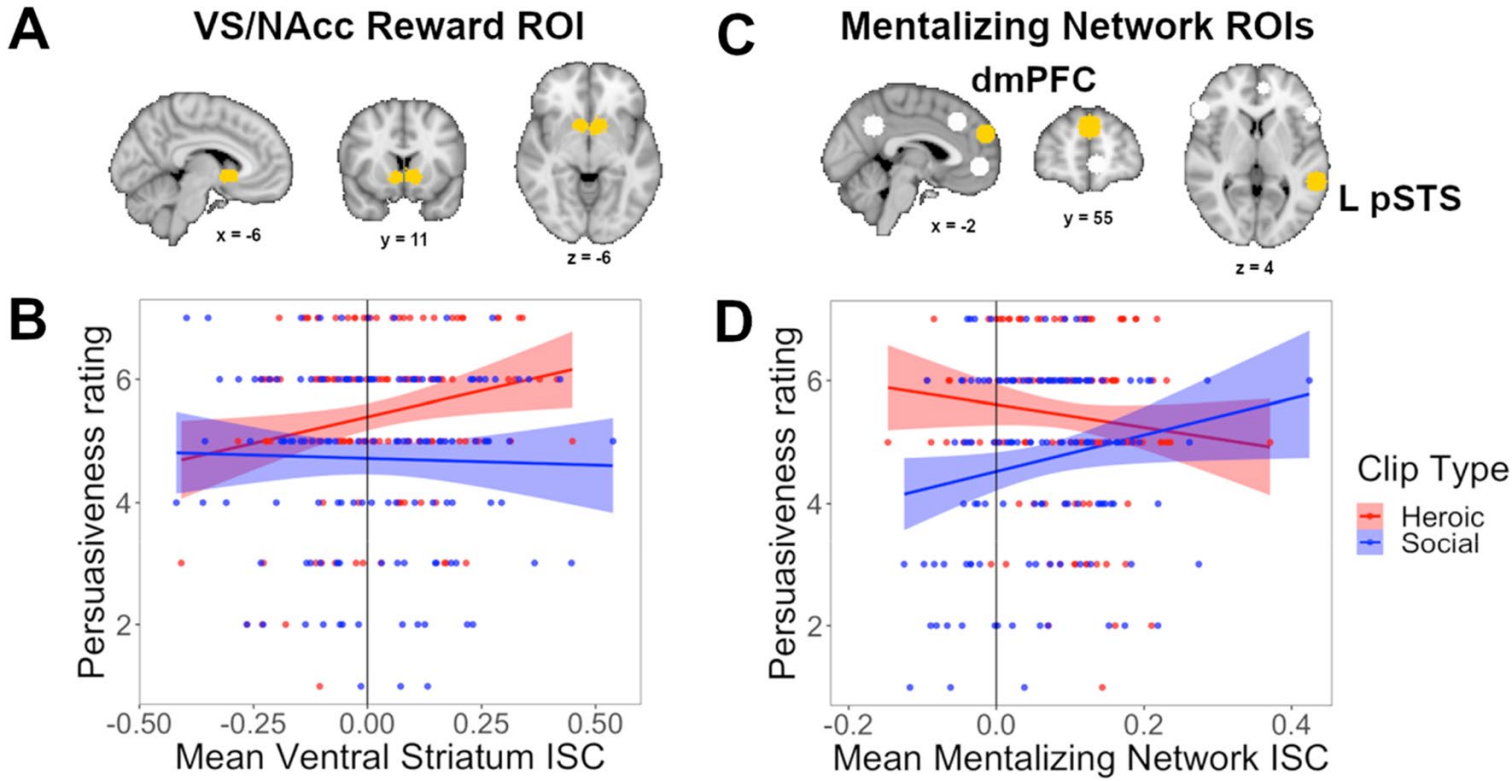
Bivariate relationships between ISC values and persuasiveness ratings for Heroic and Social stimuli. ISCs averaged across L and R hemispheres in ventral striatum/nucleus accumbens (VS/NAcc), displayed in yellow on panel (**A**), predict increased persuasiveness for Heroic stimuli but not for Social stimuli (**B**). Mean ISCs averaged across 14 ROIs in the mentalizing network, some of which are displayed in white or yellow on panel (**C**), particularly in dmPFC and L pSTS (highlighted in yellow on panel (**C**), predict increased persuasiveness for Social stimuli but not for Heroic stimuli (**D**).

After identifying an optimal behavioral model, a key analysis of interest was the extent to which neural measures predicted additional variance in persuasiveness ratings. The goal of this analysis was to find evidence via neural measures for the presence of psychological mechanisms predicted to be involved in persuasion but not captured by behavioral measures. Brain regions associated with reward were of particular interest based on prior literature. ROIs were defined following^22^. Intersubject correlations (ISCs) were computed in right (R) and left (L) ventral striatum/nucleus accumbens (VS/NAcc) ROIs, which are key nodes in the reward-sensitive circuit strongly associated with preferences (Fig. 1A). When adding ISCs in VS/NAcc, averaged across hemispheres, to the base model shown in Table 1, three different model specifications were tested: main effects only, interaction with stimulus type, and interactions with both stimulus type and participant gender. The model including an interaction with stimulus type yielded the lowest AIC value. An ANOVA comparing this model to the base model (the behavioral model shown in Table 1) showed that adding ISC values for VS/NAcc predicted significantly more variance than the base model (χ^2^ = 7.02, df = 2, *p* = 0.030). Both a main effect of VS/NAcc (β = 0.109, *t* = 2.01, *p* = 0.045) and an interaction between VS/NAcc ISC and stimulus type (β = 0.118, *t* = 2.13, *p* = 0.034) were observed on persuasiveness ratings. Separating the analyses by condition to examine simple effects found that VS/NAcc ISC was a significant positive predictor of persuasiveness for Heroic videos (β = 0.220, *t* = 2.81, *p* = 0.006), but not for Social videos (β = − 0.030, *t* = − 0.36, *p* = 0.72). Figure 1B shows simple correlations between VS/NAcc ISC and persuasiveness, which were similarly significant for Heroic clips (*r* = 0.21, *p* = 0.015) but not for Social clips (*r* = − 0.03, *p* = 0.76). Thus, greater alignment in fluctuations of VS/NAcc activity across individuals predicted an increase in rated persuasiveness, specifically for Heroic clips.

This effect appears to be specific to VS/NAcc, rather than extending throughout the reward network. For ISCs averaged across R and L vmPFC, the model with the lowest AIC included an interaction with stimulus type, but this model predicted only marginally more variance than the base model (χ^2^ = 4.89, df = 2, *p* = 0.087). The model showed no main effect of vmPFC ISC (β = 0.043, *t* = 0.82, *p* = 0.41) but did show a marginal interaction with stimulus type (β = − 0.095, *t* = − 1.85, *p* = 0.065). Simple effects did not show a reliable effect either in the Heroic condition (β = − 0.065, *t* = -0.90, *p* = 0.37) nor in the Social condition (β = 0.127, *t* = 1.60, *p* = 0.113). Finally, when examining ISCs averaged across R and L ventral tegmental area (VTA) defined according to^41^, adding only main effects to the base model produced a lower AIC value than models with any interaction terms, but this model still did not differ from the base model in predictive power (χ^2^ = 0.01, df = 1, *p* = 0.94).

### Predicting persuasion by mentalizing-related brain activity

Both activity and ISC in brain regions that belong to the mentalizing network have been demonstrated in prior work to predict persuasiveness, and so these regions form another network of a priori interest. The strongest result emerged when averaging ISCs across a set of 14 ROIs defined by activation peaks from a recent meta-analysis of mentalizing (Fig. 1C)^21^. Here again, adding ISC values as well as an interaction term for stimulus type yielded the lowest AIC value. This model predicted significantly more variance than the base model (χ^2^ = 13.88, df = 2, *p* < 0.001). A main effect of mentalizing network ISC was marginal (β = 0.090, *t* = 1.66, *p* = 0.099), and an interaction between mentalizing network ISC and stimulus type was significant (β = − 0.169, *t* = -3.18, *p* = 0.0016). Simple effect analyses showed that mentalizing network ISC was a significant predictor of persuasiveness for Social clips (β = 0.258, *t* = 3.08, *p* = 0.0026), but not for Heroic clips (β = − 0.059, *t* = -0.81, *p* = 0.42). Figure 1D shows the simple correlation between mean mentalizing network ISC and persuasiveness, which was significant for Social clips (*r* = 0.18, *p* = 0.038) but not Heroic clips (*r* = -0.12, *p* = 0.18). Thus, the degree to which fluctuations in activity throughout the mentalizing network aligned across individuals predicted higher rated persuasiveness for Social clips.

These effects appear to be driven most strongly by ROIs in dmPFC and L pSTS, highlighted in yellow in Fig. 1C. A model including the main effect of ISC in dmPFC and its interaction with stimulus type predicted significantly more variance than the base model (χ^2^ = 13.50, df = 2, *p* = 0.001). Here, the main effect of dmPFC ISC was significant (β = 0.171, *t* = 3.28, *p* = 0.001), but with no interaction between dmPFC and stimulus type (β = − 0.052, *t* = − 0.99, *p* = 0.32). Breaking down the analysis by condition, to allow comparison with simple effects from other analyses, shows that the effect was significant for Social stimuli (β = 0.231, *t* = 2.96, *p* = 0.004) and was marginal in the same direction for Heroic stimuli (β = 0.123, *t* = 1.74, *p* = 0.085). For L pSTS, a model including both a main effect of ISC and an interaction with stimulus type again predicted significantly more variance than the base model (χ^2^ = 7.78, df = 2, *p* = 0.020). This model did not show a main effect of L pSTS ISC (β = 0.062, *t* = 1.21, *p* = 0.23) but there was a significant interaction with stimulus type (β = -0.121, *t* = -2.38, *p* = 0.018). Simple effects confirmed a significant effect for Social stimuli (β = 0.184, *t* = 2.33, *p* = 0.021) but not for Heroic stimuli (β = -0.073, *t* =− 1.04, *p* = 0.30). Adding ISCs in each of the other 12 individual ROIs from the meta-analysis^21^ along with their interaction with stimulus type did not significantly improve predictive power relative to the base behavioral model, with uncorrected p < 0.05. Note that the effect in dmPFC survived FDR correction for multiple comparisons across all 14 regions (corrected p = 0.016), while the effect in L pSTS did not (corrected p = 0.14).

The relationship between ISCs in the mentalizing network and rated persuasiveness of Social clips was not limited to a particular definition of this network. When ISCs in the mentalizing network were computed by averaging across 9 ROIs defined via a different meta-analysis^42^, a model that added ISCs and their interaction with stimulus type to the behavioral model also significantly improved predictive power (χ^2^ = 7.20, df = 2, *p* = 0.027). This model showed an interaction between mentalizing network ISCs and stimulus type (β = − 0.118, *t* = − 2.20, *p* = 0.029) but no main effect (β = 0.064, *t* = 1.17, *p* = 0.24). Breaking the results down by stimulus type showed a significant effect for Social stimuli (β = 0.192, *t* = 2.39, *p* = 0.018) but not Heroic stimuli (β = − 0.042, *t* = -0.55, *p* = 0.58). The same result was observed using a mentalizing network defined by averaging across 12 mentalizing-related activations in a specific task that was intended as a more precise way of defining the mentalizing network than meta-analyses that combine data from varied mentalizing tasks^43^. Here again, a model with ISCs and their interaction with stimulus type significantly improved predictive power (χ^2^ = 11.02, df = 2, *p* = 0.004). This model showed an interaction between mentalizing network ISC and stimulus type (β = − 0.159, *t* = -2.96, *p* = 0.003) but no main effect (β = 0.068, *t* = 1.27, *p* = 0.20). Breaking this analysis down by stimulus type showed a significant effect for Social stimuli (β = 0.212, *t* = 2.64, *p* = 0.009) but not Heroic stimuli (β = − 0.073, *t* = − 0.96, *p* = 0.34).

### Relationship between reward and mentalizing on prediction

Another relevant question in light of prior work (e.g.,^16^) is whether the relationships between persuasion and ISCs in reward and mentalizing circuits were independent from each other. A regression model including terms for both ISC in VS/NAcc (reward region) and mean ISC across the mentalizing network defined by^21^, as well as interactions between each type of ISC and clip type, had lower AIC (687.77) than models that included only reward (698.45) or only mentalizing (691.58). This analysis showed a main effect of VS/NAcc ISC (β = 0.106, *t* = 2.01, *p* = 0.045) but not mentalizing network ISC (β = 0.084, *t* = 1.57, *p* = 0.12). More importantly, as was the case when the two networks were analyzed separately, there was an interaction between mentalizing network ISC and stimulus type (β = -0.176, *t* = − 3.35, *p* < 0.001), as well as an interaction between VS/NAcc ISC and stimulus type (β = 0.127, *t* = 2.34, *p* = 0.020). Breaking the analysis down by stimulus type showed, for Heroic clips, an effect of VS/NAcc ISC (β = 0.226, *t* = 2.88, *p* = 0.005) but no effect of mentalizing network ISC (β = -0.070, *t* = -0.96, *p* = 0.34). Social clips, by contrast, showed an effect of mentalizing network ISC (β = 0.257, *t* = 2.99, *p* = 0.003) but no effect of VS/NAcc ISC (β = -0.041, *t* = -0.50, *p* = 0.62). These results suggest that reward and mentalizing had independent and dissociable effects on persuasiveness.

### Control for low-level features

A possible alternate explanation for our results is that differences between videos in low-level visual or auditory stimulus features are responsible for the observed relationships between ISCs and persuasion. Follow-up analyses were run to address this issue. For each video clip, time series were estimated for four visual features (brightness, saliency, sharpness, vibrance) and two auditory features (spectral centroid and root mean square of the auditory signal). These six time series were regressed out from the time series of left and right VS/NAcc and from each node in the mentalizing network. ISC analyses were recomputed based on the residuals from these regressions. The primary results were still observed in this analysis. In VS/NAcc, mean ISC predicted persuasiveness ratings, with a main effect (β = 0.111, *t* = 2.05, *p* = 0.041) and an interaction between ISC and stimulus type (β = 0.121, *t* = 2.20, *p* = 0.028). More specifically, ISC predicted persuasiveness for Heroic videos (β = 0.227, *t* = 2.93, *p* = 0.004) but not Social videos (β = − 0.032, *t* = − 0.38, *p* = 0.71). In the mentalizing network, there was a marginal main effect of ISC on persuasiveness (β = 0.100, *t* = 1.84, *p* = 0.068) and an interaction between ISC and stimulus type (β = -0.188, *t* = − 3.54, *p* < 0.001). Mentalizing network mean ISC predicted persuasiveness ratings for Social videos (β = 0.275, t = 3.20, p = 0.002) but not for Heroic videos (β = − 0.067, t = -0.90, p = 0.37). The finding that these relationships are, if anything, stronger than those computed from the raw data enhances confidence that observed effects emerged due to high-level features, rather than resulting from differences in low-level features between videos.

### Predicting persuasion by whole-brain activity

As an exploratory analysis, the degree to which ISCs in pre-defined parcels added predictive value to models predicting persuasiveness was examined in a set of 300 parcels assigned to 7 networks, defined according to a whole-brain parcellation^44^. When averaging ISCs across all parcels in a given network, and modeling both the main effect of ISC and its interaction with stimulus type, only the Default network improved prediction of the model at an uncorrected p < 0.05 threshold. The model including both mean Default network ISC and its interaction with stimulus type predicted significantly more variance than the base model (χ^2^ = 9.73, df = 2, *p* = 0.0077). However, this effect was just below the threshold for significance after FDR correction for multiple comparisons (p = 0.054). The model yielded both a main effect of ISC (β = 0.111, *t* = 2.06, *p* = 0.04) and an interaction with stimulus type (β =− 0.105, *t* = -1.99, *p* = 0.047). Breaking down the model by stimulus type showed a significant effect for Social stimuli (β = 0.214, *t* = 2.69, *p* = 0.008) but not for Heroic stimuli (β = 0.013, *t* = 0.17, *p* = 0.86). Thus, greater alignment across individuals of fluctuations in Default network activity predicted higher rated persuasiveness for Social stimuli, similar to the results reported for the mentalizing network.

**Figure 2:**
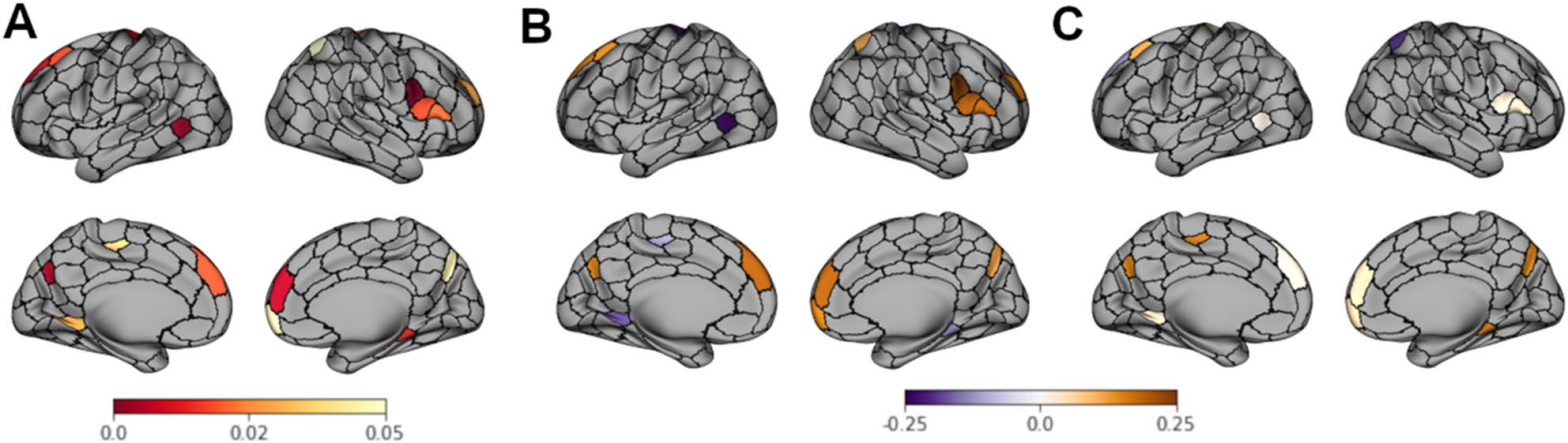
Exploratory parcel-based analysis showing where, across the whole brain, ISC values add to the predictive power of a behavioral model predicting persuasiveness. (**A**) p values (not corrected for multiple comparisons) reflecting increased predictive power when ISC in a particular region, and its interaction with stimulus condition, are added to the model. (**B**,**C**) β coefficients indicating the direction and strength of the relationship between ISC values and persuasiveness, in all parcels with p < .05 in panel (**A**), for (**B**) Social and (**C**) Heroic stimuli.

Figure 2A shows analogous results broken down by individual parcel. Specifically, the degree to which predictive value increased significantly relative to the base behavioral model when ISCs in individual parcels, and their interaction with stimulus condition, were included in the model, is shown for all parcels in which uncorrected p < 0.05. None of these effects were strong enough to survive FDR correction for multiple comparisons across all 300 parcels but are reported on an exploratory basis. Coefficients representing effects in the parcels showing an effect in Fig. 2A were computed and plotted separately for Social (Fig. 2B) and Heroic (Fig. 2C) stimuli. Regions associated with mentalizing, including dmPFC, right inferior frontal gyrus, and precuneus, were among those that showed evidence of added predictive value, particularly for Social stimuli.

### Aggregate ISC differences by clip condition

An alternative approach to examining these data is to compare how ISCs differed between conditions when data from all stimuli of a given type are averaged together. These analyses address how processing of the two types of stimuli differ on average. Similar analyses comparing intersubject functional connectivity (ISFC) between Heroic and Social stimuli are reported in Supplemental Material. Paired-samples permutation tests with 100,000 permutations were used to compare each individual’s mean ISC score for the 4 Heroic videos vs. the 4 Social videos. Mean ISCs were higher for Heroic videos when averaging ROIs across the mentalizing network, whether defined according to the set of activations from^21^ (*p* = 0.005), ^42^ (*p* = 0.019), or ^43^ (*p* = 0.006). However, no difference between conditions was apparent for the two specific mentalizing regions from^21^ cited above as having most strongly predicted persuasiveness in Social videos: dmPFC (uncorrected *p* = 0.79) and L pSTS (uncorrected *p* = 0.58). With respect to reward ROIs, the VS/NAcc ROI showed no aggregate difference in ISCs by condition (uncorrected *p* = 0.72), nor did ROIs for vmPFC (uncorrected *p* = 0.43) or VTA (uncorrected *p* = 0.48).

**Figure 3:**
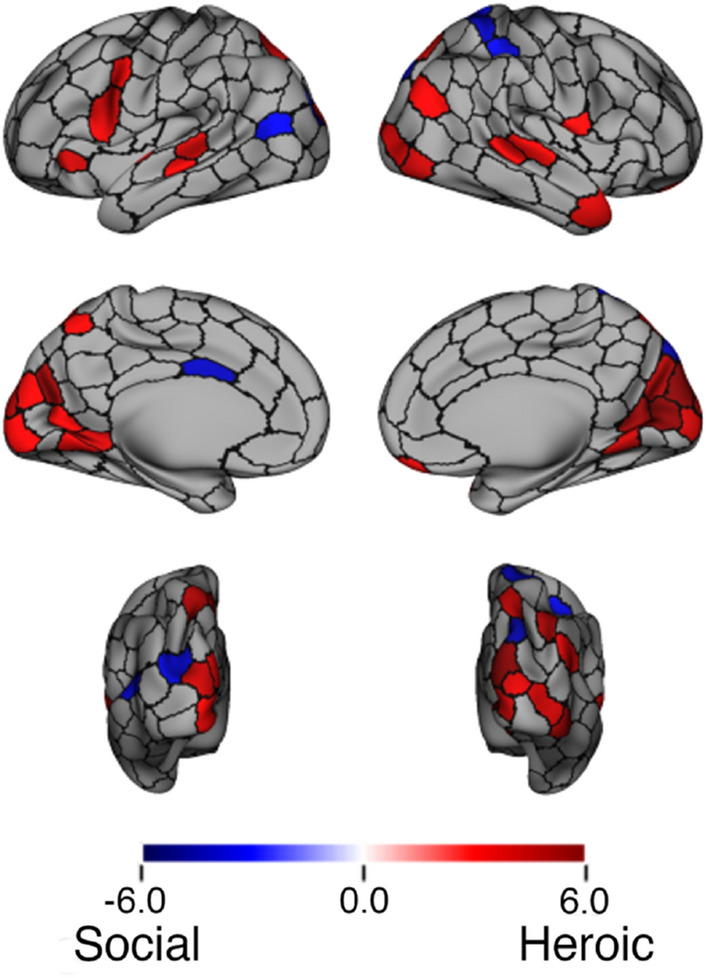
Parcels showing a difference in mean ISC by stimulus condition. Colors represent the *t *statistic for the difference in mean ISC value by condition. The map is thresholded based on parcels having corrected p < .05 following FDR correction on a paired permutation test.

A parcel-based analysis similar to that described above showed a number of parcels in which mean ISCs differed between Heroic and Social clips. The t-statistics for all regions with a significant difference in mean ISC by condition are plotted in Fig. 3, thresholded using a paired permutation test statistic (100,000 permutations) with FDR correction applied for multiple comparisons across all 300 parcels. Aggregating parcels by network, Heroic clips had higher ISCs in 4 of 7 networks, with FDR correction applied across the 7 networks: Visual (corrected *p* = 0.002), Dorsal Attention (corrected *p* = 0.023), Salience/Ventral Attention (corrected *p* = 0.034), and Default (corrected *p* = 0.003). There were no significant differences by condition in the Somatomotor (corrected *p* = 0.28), Limbic (corrected *p* = 0.22), or Control (corrected *p* = 0.09) networks.

## Discussion

Greater ISCs in VS/NAcc predicted rated persuasiveness of Heroic videos but not of Social videos. Greater ISCs in brain regions associated with mentalizing, including most notably dmPFC, predicted rated persuasiveness of Social videos but largely not of Heroic videos. ISCs averaged across Default network parcels were similarly positively associated with persuasiveness of Social videos but not Heroic videos. Heroic videos in the aggregate tended to produce larger ISCs than Social videos across visual, attention, and default network brain regions, potentially consistent with Heroic videos eliciting greater arousal than Social videos (cf.^45^). Finally, behavioral results were largely consistent with prior work as Heroic videos tended to be rated as more persuasive, and narrative transportation also predicted persuasiveness independently from the effects computed from neural measures.

Together, these results provide insight into the psychological mechanisms by which extremist propaganda persuades an audience. They specifically support the hypothesized distinction between Heroic and Social narrative structures. The functions of the specific brain regions in which ISCs were differentially associated with persuasiveness for the two types of narratives fit well with how those narrative categories have been conceptualized^3^. Heroic martyr narratives emphasize one’s prospect of personal glory as an individual who achieves extraordinary feats for the group based on that person’s personal capabilities. Increased activity in VS/NAcc has been associated in prior literature with preferences in a variety of domains, all of which could be perceived as producing positive affect in anticipation of something pleasurable. It follows that Heroic-narrative videos aiming to persuade via a focus on personal glory would be judged as most likely to do so when they modulate brain activity in this region consistently across individuals, as our neuroimaging results show. Social martyr narratives, in contrast, are defined by the prospective martyr empathizing with those within the community who suffer, prompting a desire to alleviate the suffering of the community with an act of individual sacrifice. It emphasizes identity with the group and encourages people to place a group identity above their personal identity^46^. This definition connects with prior studies in which activity in dmPFC predicts persuasiveness in response to negative information (e.g.^27,29^), with work associating ISCs in dmPFC with negative emotional valence^45^, and with the general function of the mentalizing network in considering what other people are thinking and feeling (e.g.^21^). Our finding that intersubject correlations in these regions predict rated persuasiveness for Social-narrative videos supports the hypothesized psychological mechanisms behind the effectiveness of this type of propaganda.

Our study is the first to show a dissociation between how brain measures from reward/valuation regions and measures from mentalizing circuitry separately predict persuasion based on the narrative content of the stimuli. The finding that reward and mentalizing circuits can have dissociable effects on persuasion is consistent with some prior work (e.g.^29^). Similar results have also been observed in a content sharing task via effects of an instructional mindset manipulation on brain activity^47^. Specifically, in that study, activity in the reward circuit only reliably increased relative to the control task when participants were instructed to choose content with a motivation to “Describe Yourself”, which has a similar self-oriented focus as our Heroic narratives. Approaching the sharing task with the mindset to “Help Somebody” can be considered analogous to the community-oriented motivations evoked by our Social narratives. This framing was associated with increased activity in Self-focused and Mentalizing circuitry relative to the control task, with the “Describe Yourself” manipulation also increasing activity in these regions. These results can be interpreted as being in alignment with the results of the present study.

Some potential limitations to the external validity of our results need to be acknowledged. Participants were recruited from among University of Chicago students and the surrounding community. Additionally, as described in Methods, none of our participants identified as Muslim, and nearly half had no stated religion. Our sample also leaned heavily liberal. Thus, this sample is likely not representative of the target audience for such videos. An interesting question for future research would be whether the mechanisms of persuasion for extremist messaging differ based on the degree to which an audience member’s own political views align with the content being viewed.

Another limitation is that we were not able to quantify the relative real-world effectiveness of these videos. ISIS does not share view counts publicly, and the videos are typically released in a decentralized manner across social media platforms and are often removed by content moderators and reposted from new accounts, making it very difficult to reliably estimate total engagement. Additionally, the number of videos in each category was not sufficient to robustly relate brain measures to out-of-sample measures of persuasion or engagement. Thus, we do not have direct evidence that ISC can predict these metrics outside of the current sample. It is clear, however, that these videos do play a role in recruiting fighters from Western countries based on surveys of ISIS recruits indicted for terrorism crimes in the United States^1^. Additionally, increased activity in the brain regions showing the strongest effects here, specifically VS/NAcc and dmPFC, have been associated with real-world engagement and persuasion in many prior studies (e.g.,^16–18,25–27^). Our results show that consistent modulation of activity in these regions predicts persuasiveness. It is reasonable to assume that stimuli aiming to recruit extremists could be also more persuasive in the real world when they consistently modulate brain activity in these regions. Further work will be necessary to test this possibility more directly.

A related limitation is that the specific pattern of results reported here was not predicted a priori, though it is consistent with prior literature. Additionally, some analytic flexibility was leveraged in exploratory analyses to determine the most meaningful result. Thus, while the results are robust within this sample, we cannot be certain that these results apply beyond this particular sample or to other similar persuasive materials. Further work will be needed to replicate and extend the findings reported here.

Another complication is how to interpret the results in vmPFC. Both VS/NAcc and vmPFC have been identified as core regions in the brain’s reward circuitry and value system^22^, but higher ISCs in vmPFC were associated with, if anything, slightly higher persuasiveness ratings for Social stimuli, not Heroic stimuli. This would be more consistent with our findings in mentalizing regions such as dmPFC than with effects in VS/NAcc. Still, there are differences in the roles of VS/NAcc and vmPFC. For instance, in a study of crowdfunding campaigns, reward-related activity in both NAcc and mPFC predicted preferences within individuals, but only NAcc activity predicted out-of-sample campaign success^17^. This finding is consistent with the idea that mPFC integrates a wider range of inputs to decision-making, which could include idiosyncratic preference factors, while VS/NAcc activity reflects a more primary reward response^48^. Thus, fluctuation in vmPFC driven by reward might reflect more idiosyncratic factors than reward-driven fluctuations in VS/NAcc, and would not modulate ISCs. Additionally, vmPFC is associated with mentalizing as well as with reward (e.g.,^21^), so the effects that are present in vmPFC may relate more to its role in mentalizing. It is therefore reasonable to interpret ISCs in VS/NAcc as reflecting more of a pure reward signal, while the role of vmPFC is more complex to interpret.

Ultimately, our results provide novel insight both on the specific topic of ISIS recruitment methods and more broadly on the neuroscience of persuasion and political propaganda. While some other studies have associated ISCs in the mentalizing network with popularity and persuasiveness of naturalistic stimuli, this work is, to our knowledge, the first to relate ISCs measured in a core reward-sensitive brain region to persuasiveness. It also provides novel evidence as to how reward/value and mentalizing networks work either independently or in concert to achieve persuasion. Finally, this work provides key support for the hypothesis that ISIS recruitment materials employ two distinct psychological routes to achieve persuasion^4^. Persuasiveness of Heroic clips, which have been shown to appeal specifically to people without strong ties to the Muslim community and individuals who are looking for personal glory, is associated with ISCs in a key node of the reward circuit. Persuasiveness of Social clips, which focus on threats to the Muslim community and appeal more to people with stronger ties to that community, is associated with ISCs in mentalizing and Default networks. While we cannot be certain that the results generalize beyond the particular conditions of this study, if they did replicate in future work, these findings would imply that a variety of approaches are required to counter propaganda for antisocial causes when messaging relies on a diversity of communicative appeals. The neural metrics used here could be relied upon to evaluate the effectiveness of counter-propaganda techniques in the future. This work can be considered to be one piece of a broader effort to fight propaganda, and to make it more difficult for extremists to recruit people to participate in violent and antisocial behavior regardless of the political goal.

## Methods

### Participants

Participants were recruited from the University of Chicago student population and from the surrounding community in Chicago. All participants signed informed consent forms prior to beginning the study. A total of 49 individuals completed the study. Of these, two were excluded due to having high levels of motion and low data quality on 3 out of 4 runs. Low data quality was defined, using output from MRIQC v22.0.6^49^, as having greater than 5% of volumes with framewise displacement (FD) > 0.9 mm and being at least 2 SD worse than the mean on tSNR, AFNI quality index, and mean FD. No other participants had more than one run meeting any of these criteria. An additional participant was excluded due to a screening failure: using a medication, Adderall, that was established as an exclusion criterion. Note that participants taking psychoactive medications were allowed in the study if they only reported taking a single SSRI anti-depressant. 46 participants thus contributed some data to the analyses. Of these, 34 individuals (mean age = 21.7 years, age range = 18–48 years, 14 male, 19 female, 1 non-binary) completed the full protocol. Three participants were missing post-scan questionnaire data for one video each; data from these videos were excluded from the primary data but included in the aggregate data. Of the 34 primary participants, 16 reported their religious background as atheist/agnostic/unaffiliated, 10 Christian, 4 Jewish, 1 Buddhist, 1 Hindu, 1 who reported multiple religions, and 1 who did not respond. Politically, 30 reported leaning towards the Democratic Party, with two leaning weakly Republican and 2 independents. Data from the first 12 participants (mean age = 26.8 years, age range = 20–42, 4 male, 8 female), who were scanned while watching the videos but did not complete the post-scan questionnaire, were included in aggregate data but excluded from other analyses. The first 10 participants also had a slightly different timing configuration, as noted below.

### Behavioral procedure

All procedures were approved as minimal risk by the University of Chicago Social and Behavioral Sciences (SBS) Institutional Review Board (IRB). Research protocols were in accordance with the Declaration of Helsinki and with the U.S. federal Common Rule (45 CFR part 46) regulating human subjects research. Video clips were presented across four scan runs, with two videos (one Heroic and one Social) assigned to each run, in random order. Each run began with 4 s of fixation after the initial magnetic stabilization. Between the two videos, a fixation cross was presented lasting either 4 s (first 10 subjects) or 16 s (final 36 subjects). Additional rest time was also present at the end of each scan run, which differed based on the length of the specific videos in that run. The duration of each run was 126 s (first 10 subjects) or 148 s (final 36 subjects).

Videos overlapped with those described in our previous work on this topic^5^. Specifically, the present study used videos labeled H01, H03, H06, and H07 for the Heroic condition and videos labeled S01, S02, S05, and S06 for the Social condition. Heroic videos lasted 49 s, 60 s, 63 s, and 61 s, while Social videos lasted 34 s, 55 s, 38 s, and 54 s. The videos were selected from an initial set of 36 videos (18 ostensible Heroic videos and 18 ostensible Social videos) based on a pilot study in which 79 adults rated all 36 clips on whether they appealed to duty/obligation (Social) or glory/self-empowerment (Heroic)^5^. The 14 videos (7 Heroic, 7 Social) with the most extreme ratings on either end of the scale were selected for inclusion in that study. In this larger set of videos, it was confirmed that YIQ luminance values did not differ as a function of narrative type (Heroic vs. Social). The 8 videos used in the current work were a subset of the 14 videos selected for that study. For the present work, four visual features (brightness, saliency, sharpness, vibrance) and two auditory features (spectral centroid and root mean square of the auditory signal) were quantified for each video; the average scores on these metrics also did not reliably differ between Heroic and Social videos.

As part of the pre-scan screening procedure, participants completed an 8-item Justice Sensitivity measure^39^ and the 5-item DUREL religiosity index^40^. In the scanner, the video clip task described presently was preceded by an unrelated moral reasoning task similar to^50^; fMRI data from that task will be reported elsewhere. As part of the pre-scan screening procedure, prospective participants were only recruited if they showed some strong opinions on a set of sociopolitical issues used in the separate moral reasoning task. After the scan, participants were given the opportunity to watch each video again. Immediately after watching each video, they were asked to answer 4 questions on a 7-point Likert scale: “This video would help the militant group recruit” (Persuasiveness), “I could picture myself in the scene of the events described in the video.” (Narrative transportation 1), “After the video ended, I found it easy to put it out of my mind.” (Narrative transportation 2, reverse-scored), and “I found my mind wandering while watching the video.” (Narrative transportation 3, reverse-scored). Scores on the final 3 questions were averaged to generate a measure of narrative transportation for each video.

### Neuroimaging procedure

Functional MRI data were collected on a Philips Achieva 3 T MRI scanner at the University of Chicago MRI Research Center. Functional scans used a 2000 ms TR length, 28 ms TE length, flip angle 80°, 40 ascending slices with a 0.3 mm gap between slices. Voxel size was 3.0 mm × 3.1 mm × 3.0 mm, with a 64 × 62 matrix, and field of view of 192 × 192 mm^2^. Scans for the first 10 participants had 63 volumes, while scans for the final 36 participants had 74 volumes. A T1-weighted structural image was also collected, with TR length = 8 ms, TE length = 3.5 ms, flip angle 8°, 0.85 mm × 0.85 mm × 0.85 mm voxels, and a 284 × 260 matrix. For field inhomogeneity mapping, two short runs were collected using the same parameters as the functional runs, with 5 volumes collected in the anterior → posterior direction and 5 volumes collected in the posterior → anterior direction.

### fMRI Preprocessing

Data were preprocessed using fmriprep v22.1.1^51^. The following 4 paragraphs are excerpted and adapted from the documentation distributed with fmriprep. The T1-weighted (T1w) image was corrected for intensity non-uniformity (INU) with N4BiasFieldCorrection^52^, distributed with ANTs 2.3.3 ^53^, and used as T1w-reference throughout the workflow. The T1w-reference was then skull-stripped with a *Nipype* implementation of the antsBrainExtraction.sh workflow (from ANTs), using OASIS30ANTs as target template. Brain tissue segmentation of cerebrospinal fluid (CSF), white-matter (WM) and gray-matter (GM) was performed on the brain-extracted T1w using fast (part of FSL 6.0.5.1)^54^. Brain surfaces were reconstructed using recon-all (FreeSurfer 7.2.0)^55^, and the brain mask estimated previously was refined with a custom variation of the method to reconcile ANTs-derived and FreeSurfer-derived segmentations of the cortical gray-matter of Mindboggle^56^. Volume-based spatial normalization to standard space was performed through nonlinear registration with antsRegistration (ANTs 2.3.3), using brain-extracted versions of both T1w reference and the T1w template. The following template was used for spatial normalization: *FSL’s MNI ICBM 152 non-linear 6th Generation Asymmetric Average Brain Stereotaxic Registration Model* (TemplateFlow ID: MNI152NLin6Asym)^57^. A *B*_*0*_-nonuniformity map (or *fieldmap*) was estimated based on two echo-planar imaging (EPI) references with topup (FSL 6.0.5.1)^58^.

For each BOLD run, the following preprocessing was performed. First, a reference volume and its skull-stripped version were generated using a custom methodology of *fMRIPrep*. Head-motion parameters with respect to the BOLD reference (transformation matrices, and six corresponding rotation and translation parameters) were estimated before any spatiotemporal filtering using mcflirt (FSL 6.0.5.1)^59^. The estimated *fieldmap* was then aligned with rigid-registration to the target EPI (echo-planar imaging) reference run. The field coefficients were mapped on to the reference EPI using the transform. BOLD runs were slice-time corrected to 0.975 s (0.5 of slice acquisition range 0 s-1.95 s) using 3dTshift from AFNI^60^. The BOLD reference was then co-registered to the T1w reference using bbregister (FreeSurfer) which implements boundary-based registration^61^. Co-registration was configured with six degrees of freedom.

A set of physiological regressors were extracted to allow for component-based noise correction (a*CompCor*)^62,63^. Principal components were estimated after high-pass filtering of the *preprocessed BOLD* time-series (using a discrete cosine filter with 128 s cut-off). Probabilistic masks for CSF and WM were generated in anatomical space, and components were calculated separately within each mask.

The BOLD time-series were resampled into standard space, generating a preprocessed BOLD run in MNI152NLin6Asym space. First, a reference volume and its skull-stripped version were generated using a custom methodology of *fMRIPrep*. All resamplings can be performed with a single interpolation step by composing all pertinent transformations (i.e. head-motion transform matrices, susceptibility distortion correction when available, and co-registrations to anatomical and output spaces). Gridded (volumetric) resamplings were performed using antsApplyTransforms (ANTs), configured with Lanczos interpolation to minimize the smoothing effects of other kernels^64^. Non-gridded (surface) resamplings were performed using mri_vol2surf (FreeSurfer).

### fMRI data analysis

A selection of the confound regressors obtained from fmriprep were applied to the preprocessed data using the Nilearn clean_img function. The 6 motion parameter time series derived from head motion estimates calculated in the correction step were expanded with the inclusion of temporal derivatives and quadratic terms^65^, yielding a total of 24 motion parameters (6 basic parameters plus squared, derivatives, and squared derivatives of each one). The confound model also included the first 5 aCompCor parameters from WM, and up to the first 5 aCompCor parameters from CSF, though sometimes as few as 3 CSF parameters were used if fewer than 5 components were needed to account for 50% of variance in CSF signal. A cosine basis regressor was included for high-pass filtering if available. For the first 10 participants, a high pass filter of 0.008 Hz was applied in clean_img because the run was too short to include cosine basis functions. The first volume from each run was dropped in order to allow for use of derivatives of motion parameters as regressors.

Data from each run was resampled to match the ROI space using the Nilearn resample_to_img function. The time series for each video was then extracted from the cleaned images using the Nibabel slicer function, beginning 4 volumes after stimulus onset and ending 2 volumes after stimulus offset, to account for 4 s of delay in the hemodynamic response and to remove signal related to non-specific increases in brain activation at the beginning of each video. Each ROI was applied as a mask using the Nilearn NiftiMasker function, and BOLD signal was averaged for all voxels at a given time point within the mask to yield a 1-dimensional time series for that individual and video. Each ROI was cropped using the whole-brain mask generated by fmriprep for the relevant scan run. If fewer than 10% of the voxels within an ROI were included in the whole-brain mask for a given participant and run, no data was returned and that individual was excluded from all analyses of the ROI for that video.

Intersubject correlations (ISCs) were computed as the Pearson correlation coefficient between an individual’s time series for a specific ROI and video clip and the mean time series for all 45 other individuals (or however many individuals had valid data in that ROI, if not the full sample) for the same ROI and video clip. An arctanh (Fisher’s Z) transformation was then applied to the resulting correlation coefficient.

ROIs were defined based on prior literature. For reward regions, the VS/NAcc and vmPFC ROIs were defined from a meta-analysis of domain-general reward response, as shown in Fig. 9 of^22^, obtained from www.kablelab.com. Each cluster was further separated into R and L hemisphere segments by a sagittal split at the midline. A VTA ROI was defined based on a probabilistic atlas^41^ as all voxels with at least a 10% chance of being in the VTA. This ROI was also split by hemisphere via a sagittal split at the midline. For all reward regions, ISCs were initially computed separately within each hemisphere and then averaged. To identify brain regions associated with mentalizing, spheres with radius 10 mm were constructed using *fslmaths* around reported peak coordinates from published studies. Specifically, spheres were created around all 16 peaks in Table 1 of the meta-analysis reported by^21^. A total of 14 ROIs were generated, as two pairs of spheres (in R TPJ and L precentral gyrus) overlapped, and thus these paired spheres were combined into single ROIs. For the alternative meta-analysis^42^, the peak coordinate for each of 9 clusters reported in Table 3 from studies on adults were used to create ROIs. Finally, the third definition of a mentalizing network was created from the 13 peaks (excluding cerebellum) from the Why > How contrast in Table 2 from^43^. Here, the R dmPFC sphere overlapped slightly with one of the L dmPFC spheres, so the overlapping ROIs generated from these two peaks were separated at the sagittal midline. Additionally, two other peaks in R anterior temporal cortex overlapped and were combined into a single ROI, yielding 12 total ROIs.

We used estimates of four visual features (brightness, saliency, sharpness, vibrance) and two auditory features (spectral centroid and root mean square of the auditory signal). to ensure that low-level audiovisual features were not a primary driver of our observed results, Each feature was estimated at the native video or audio resolution using the *pliers* package in Python. This time series was then converted to a 3-column file including the onset time of the video frame, the duration (0.03333 s for video, corresponding to 30 Hz; for audio, a resampled resolution of 0.4 s per instance), and the amplitude of the feature estimated by pliers. This information was then entered into the nilearn *compute_regressor* function and convolved with the Glover HRF to create a regressor. These regressors were then downsampled to 2 s using the Pandas *resample* function. The portion of each of the 6 regressors beginning 8 s after stimulus onset were extracted and entered as predictor variables in a linear mixed effects regression in R, with mean fMRI BOLD time series for VS/NAcc and mean mentalizing network for the full sample of 46 participants as the outcome variables. The residuals from each regression were extracted, and ISCs were recomputed based on these time series using the same approach as described above.

### Supplementary Information

Supplementary Information.

## Data Availability

Behavioral data, neuroimaging data extracted from regions of interest, and all analysis code are available at https://osf.io/w5qpx . Raw fMRI data have been uploaded to OpenNeuro at the following link: https://openneuro.org/datasets/ds005040/.
