# Supplementary material for: Intersubject correlations in reward and mentalizing brain circuits separately predict persuasiveness of two types of ISIS video propaganda
Source: Sci Rep. 2024 Jun 12;14:13455. doi: 10.1038/s41598-024-62341-3 (PMC11166951; doi:10.1038/s41598-024-62341-3)
Supplement: Supplementary file 1 — Supplementary Information. [file 41598_2024_62341_MOESM1_ESM.docx]

**Supplemental Material**

**ISFC analyses**

ISFC analyses are analogous to ISC analyses, but instead of examining the typicality of activity within *one* brain region, ISFC compares an individual’s activity time course in one region with the group average of activity in *other* brain regions. This approach has been validated as improving the signal-to-noise ratio relative to functional connectivity measures computed within individuals (Simony et al., 2016; Nastase et al., 2019). It is specifically useful for measuring the degree to which nodes within and across networks are simultaneously activated in response to presented stimuli, while removing noise and intrinsic connectivity effects that are unrelated to the stimulus.

For the ISFC analysis, we aggregated edges either within a network or between two specific networks. After computing p-values using permutation tests and applying FDR correction across 28 possible pairings, three pairings showed a significant difference between conditions (Figure S1A): Visual-Visual connections (corrected *p* = .029) and Visual-Dorsal Attention network connections (corrected *p* < .0001) were stronger for Social stimuli, while Visual-Default connections (corrected *p* = .028) were stronger for Heroic stimuli. As another way of representing these effects, raw connectivity matrices averaged across each of the 4 videos in each condition were submitted to a Network-Based Statistic (NBS) analysis (Zalesky et al., 2010). This analysis allows for a visualization of individual connections that differ in strength between conditions, using assumptions about the spatial distribution of edges to correct for multiple comparisons. All edges connecting to Visual network nodes and showing a difference in connectivity by stimulus condition are plotted in Figure S1B and Figure S1C. The aggregate results shown in Figure S1A motivated limiting the plots to connections with Visual nodes. Similar to what was observed in the data aggregated by network, these plots confirm that Visual nodes were broadly more likely to be coactivated with Dorsal Attention nodes as well as with other Visual nodes in the Social condition (Figure 4B) relative to the Heroic condition (Figure S1C), while the reverse was true for connections between Visual and Default network nodes.

These results indicate that brain regions that process visual stimuli were more connected to other Visual regions and to Dorsal Attention regions when processing Social videos, but visual regions were more connected to Default network regions when processing Heroic videos. They suggest an increase in attentional focus directed to Social videos, potentially consistent with the finding from prior EEG work of reduced alpha power for Social videos (Yoder et al., 2020).

Table S1. Alternate behavioral models predicting rated persuasiveness (n = 34)

|  | β | t | p |
| --- | --- | --- | --- |
| (Intercept) | -0.040 | -0.53 | 0.60 |
| Participant Gender | -0.050 | -0.66 | 0.51 |
| **Stimulus Type** | **0.257** | **4.62** | **< 0.001***** |
| Justice Sensitivity (Perpetrator subscale) | 0.103 | 1.21 | 0.24 |
| Participant Gender x Stimulus Type | 0.106 | 1.93 | 0.055 ~ |
| Justice Sensitivity (Perpetrator subscale) x Participant Gender | 0.152 | 1.90 | 0.066 ~ |
| **Justice Sensitivity (Perpetrator subscale) x Stimulus Type** | **-0.232** | **-3.70** | **< 0.001***** |
| Justice Sensitivity (Perpetrator subscale) x  Participant Gender x Stimulus Type | 0.017 | 0.30 | 0.77 |

|  | β | t | p |
| --- | --- | --- | --- |
| (Intercept) | 0.014 | 0.18 | 0.86 |
| Participant Gender | -0.052 | -0.70 | 0.49 |
| **Stimulus Type** | **0.271** | **5.26** | **< 0.001***** |
| **Narrative Transportation** | **0.346** | **5.90** | **< 0.001***** |
| Participant Gender x Stimulus Type | 0.052 | 1.02 | 0.31 |
| Narrative Transportation x  Participant Gender | -0.014 | -0.24 | 0.81 |
| **Narrative Transportation x**  **Stimulus Type** | **0.167** | **3.09** | **0.002**** |
| **Narrative Transportation x**  **Participant Gender x Stimulus Type** | **0.124** | **2.37** | **0.019*** |

Figure S1. (A) Mean difference in ISFC values by condition when connectivity for all edges within a given network or pair of networks are averaged together. Colors represent the *t* statistic for the difference in mean ISFC value by condition. (B-C) Network-based statistic results showing all individual edges with a difference in connection strength by condition, with (B) Social > Heroic and (C) Heroic > Social.


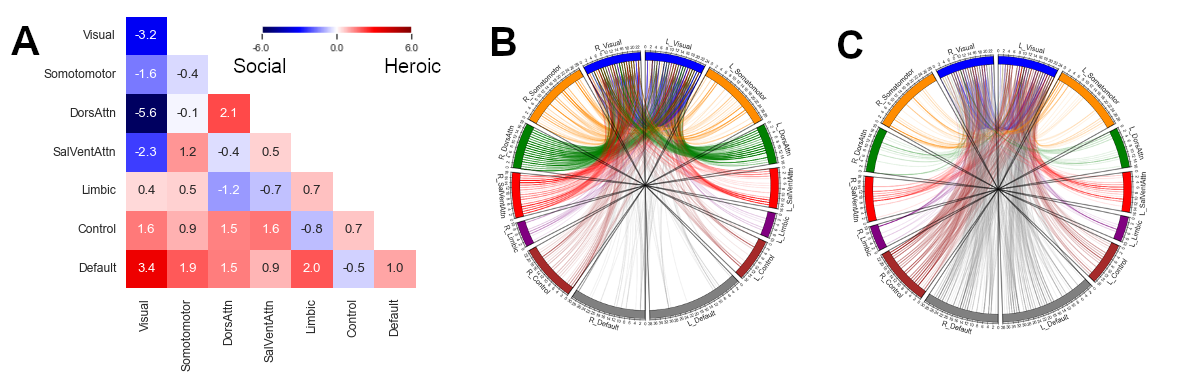


**Supplemental References**

Nastase, S. A., Gazzola, V., Hasson, U., & Keysers, C. (2019). *Social Cognitive and Affective Neuroscience, 14,* 667-685.

Simony, E., Honey, C., Chen, J., Lositsky, O., Yeshurun, Y., Wiesel, A., & Hasson, U. (2016). Dynamic reconfiguration of the default mode network during narrative comprehension. *Nature Communications, 7,* 12141.

Yoder, K., Ruby, K., Pape, R., & Decety, J. (2020). EEG distinguishes heroic narratives in ISIS online video propaganda. *Scientific Reports, 10,* 19593.

Zalesky, A., Fornito, A., & Bullmore, E. T. (2010). Network-based statistic: Identifying differences in brain networks. *NeuroImage, 53,* 1197-1207.
